# The Effect of Ultrasound, Oxygen and Sunlight on the Stability of (−)-Epigallocatechin Gallate

**DOI:** 10.3390/molecules23092394

**Published:** 2018-09-18

**Authors:** Jiajun Zeng, Huanhua Xu, Yu Cai, Yan Xuan, Jia Liu, Yue Gao, Qingxian Luan

**Affiliations:** 1 Department of Periodontology, Peking University School of Stomatology, Beijing 100081, China; zjjpanda1993@163.com (J.Z.); jessonjesson@hotmail.com (Y.C.); xuanyan-xjtu@163.com (Y.X.); dentistliujia@126.com (J.L.); 2 Department of Pharmaceutical Sciences, Beijing Institute of Radiation Medicine, Beijing 100850, China; huanhua323@163.com; 3 College of Traditional Chinese Medicine, Tianjin University of Traditional Chinese Medicine, Tianjin 300193, China

**Keywords:** green tea, stability, (−)-epigallocatechin gallate (EGCG), sunlight, oxygen, ultrasound, degradation, high performance liquid chromatography (HPLC)

## Abstract

(−)-Epigallocatechin gallate (EGCG), is the main catechin found in green tea, and has several beneficial effects. This study investigated the stability of EGCG aqueous solution under different stored and ultrasonic conditions to determine whether it can be used with an ultrasonic dental scaler to treat periodontal infection. Four concentrations (0.05, 0.1, 0.15, 2 mg/mL) of EGCG aqueous solution were prepared and stored under four different conditions (A: Exposed to neither sunlight nor air, B: Exposed to sunlight, but not air, C: Not exposed to sunlight, but air, D: Exposed to sunlight and air) for two days. The degradation rate of EGCG was measured by high performance liquid chromatography (HPLC). On the other hand, an ultrasonic dental scaler was used to atomize the EGCG solution under four different conditions (a: Exposed to neither air nor sunlight, b: Not exposed to air, but sunlight, c: Not exposed to sunlight, but air, d: Exposed to air and sunlight), the degradation of EGCG was measured by HPLC. We found that the stability of EGCG was concentration-dependent in water at room temperature. Both sunlight and oxygen influenced the stability of EGCG, and oxygen had a more pronounced effect on stability of EGCG than sunlight. The most important conclusion was that the ultrasound may accelerate the degradation of EGCG due to the presence of oxygen and sunlight, but not because of the ultrasonic vibration. Thus, EGCG aqueous solution has the potential to be used through an ultrasonic dental scaler to treat periodontal infection in the future.

## 1. Introduction

(−)-Epigallocatechin gallate (EGCG), is the main catechin found in green tea [1]. Many studies have reported its potential health benefits, including anti-inflammatory [2,3], and antioxidant actions [4] as well as the prevention of cancer and cardiovascular diseases [5,6,7] in cells, animal models, and even humans. EGCG is also known to have antimicrobial effects against both Gram-negative and Gram-positive bacteria by damaging their cell membranes, which results in the downregulation of enzymes involved in biosynthesis and the prevention of bacterial adherence to their host cell [8]. Thus, EGCG can decreases the production of toxic bacterial metabolites [8]. Based on its proven health benefits, EGCG appears to have a substantial potential for use in medical applications. 

However, EGCG is seldom used in the medical field, possibly due to its instability. In solution, EGCG donates hydrogen atoms, leading to the generation of superoxide and oxidized products. Subsequently, this causes the formation of theasinensin A and compound P2 as the major dimers, which may be the reason for the change in color of EGCG aqueous solution to brown [9]. Aqueous EGCG solution is highly unstable, as EGCG can easily degrade through oxidation and epimerization [10,11]. The rates of these two reactions are affected by many factors, such as the presence of oxygen or sunlight, as well as changes of temperature, pH and ionic strength [9,12,13]. In air-saturated buffer, EGCG undergoes auto-oxidation, forming H_2_O_2_ [11,14], while under low oxygen partial pressure, generated by extensive flushing with nitrogen gas, EGCG has shown to be stable, with 95% remaining after 6 h [15,16]. As for the pH level, the acidic environments enhance the stability of EGCG, but neutral and alkaline pH level cause auto-oxidation on the B-ring and produce theasinensin A and product P2 [17,18]. In addition, previous studies have reported that following exposure to a solar simulator emission, EGCG almost completely degrades within an hour [12,13]. The activity of EGCG is also dependent on the salt concentration of ambient medium, the degradation EGCG increases with rising ionic strength of the solvent. In addition, temperature is a significant parameter governing EGCG stability and the mode of its degradation [19,20,21]. Several works have suggested that EGCG undergoes degradation via oxidation at temperatures below 50 °C, while epimerization was more commonly occurs at higher temperatures [19,22,23]. Through epimerization, EGCG transforms to its *trans*-epimer (−)-gallocatechin gallate (GCG) [14,15,22,24], while epimerization of EGCG should not significantly alter its biological effects [25,26,27].

The stability of EGCG is affected by many factors, limiting its application in many cases; however, we hope to apply EGCG to periodontitis treatment. Periodontitis is one of the major oral inflammatory diseases, encountered in routine dentistry practice [28]. The imperative factor behind the pathogenesis of periodontal infections is the accumulation of dental plaque, which results in gingival inflammation [29]. EGCG has already been used in some clinical experiments as a mouthwash [30,31] and a local drug route [32,33,34], which showed that it has a healing effect on periodontal tissue. As ultrasonic dental scalers are widely used in the treatment of periodontitis, they are a potential delivery route for EGCG. However, before using this approach, it is necessary to investigate the stability of EGCG under ultrasound conditions to determine whether the stability of EGCG is affected by ultrasound itself, or whether it is influenced by sunlight or oxygen.

## 2. Results

### 2.1. Method Validation

#### 2.1.1. Selectivity and Specificity

**Figure 1:**
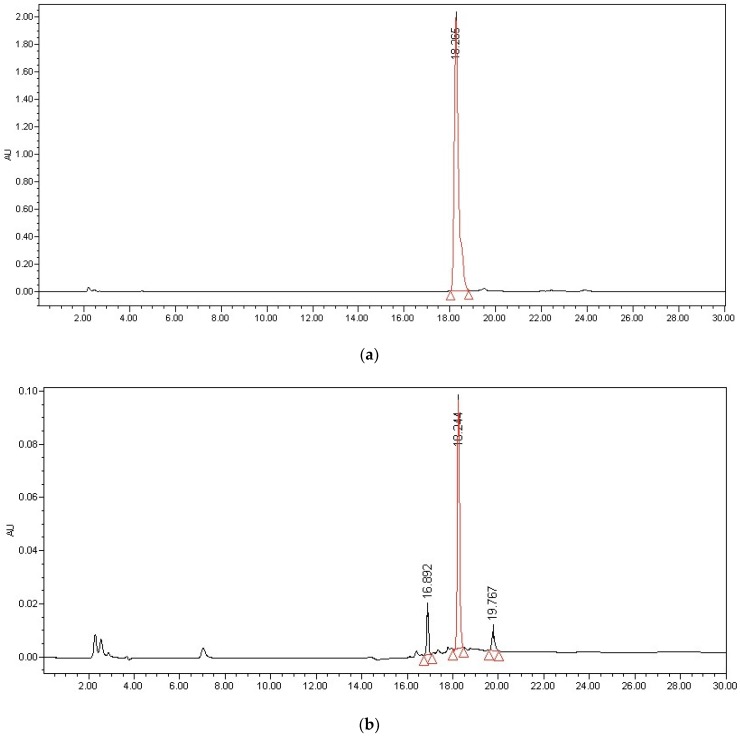
The chromatogram of the 2.00 mg/mL EGCG aqueous solution (**a**), the 0.05 mg/mL EGCG aqueous solution stored without sunlight and air at room temperature for 6 h (**b**).

The sample in the present study was EGCG aqueous solution, the retention time was 18.2 min. No endogenous interfering peaks were observed at the retention time of analytes. The chromatogram was shown in Figure 1a. In the Figure 1b, the degradation of EGCG occurred and new peaks could be detected. However, we have not fully identified the degradation products.

#### 2.1.2. Linearity and Lower Limit of Quantification (LLOQ) of EGCG in Water

The calibration curve, correlation coefficient and LLOQ were shown in Table 1. The calibration curve was constructed by plotting the peak areas versus the concentrations of EGCG in water, and it was linear over a certain range with a correlation coefficient (R^2^) larger than 0.990. The LLOQ was set to the lowest concentration on the calibration curve.

#### 2.1.3. Precision and Accuracy

The obtained intra-day/inter-day accuracy and precision data were summarized in Table 2. The intra-day accuracies were 98.88% to 99.51%, and the precisions were 1.73% and 1.93%. Meanwhile, inter-day accuracies were 93.14% and 99.85%, and the precisions were 4.19% and 1.93% respectively. The data suggested that the method was accurate and reproducible for quantification of EGCG in water.

### 2.2. Analysis of Stability of EGCG Aqueous Solution under Different Storage Conditions

**Figure 2:**
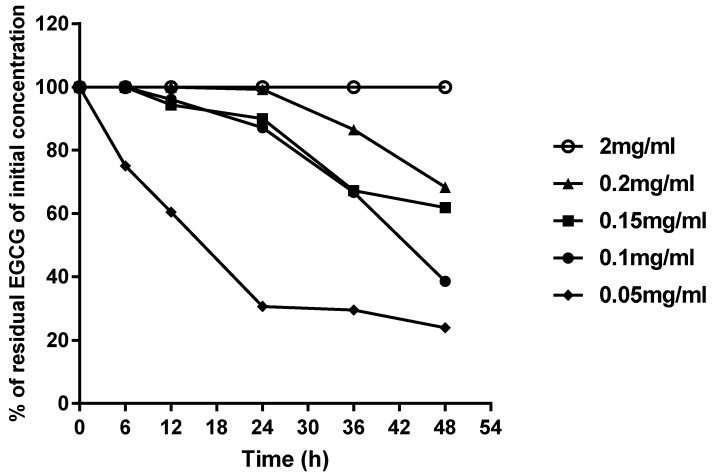
Stability of all concentrations of EGCG aqueous solutions under condition (A): exposed to neither sunlight nor air.

**Figure 3:**
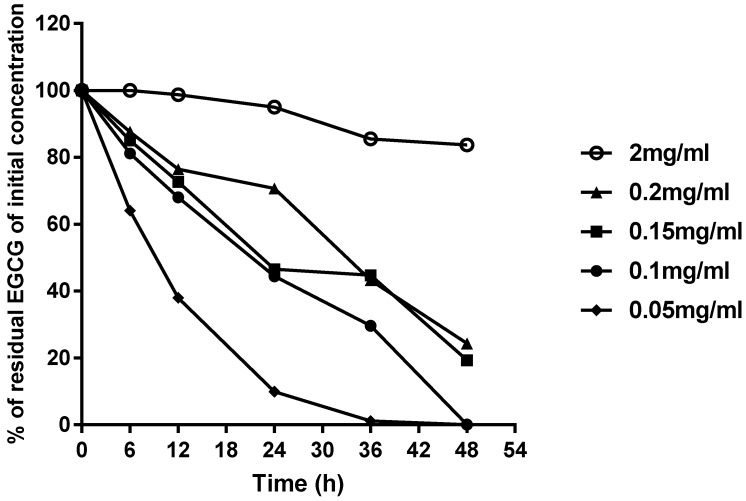
Stability of all concentrations of EGCG aqueous solutions under condition (D): exposed to sunlight and air.

The four concentrations (0.05, 0.10, 0.15, 2.00 mg/mL) used in the study cover the range of concentrations used in most animal studies [16]. Figure 2 and Figure 3 show the most stable and the most unstable conditions, respectively. The stability of EGCG increased with respect to the increase in its concentration. Under condition (A), the 2.00 mg/mL EGCG solution was rather stable after a 48-h storage period, while the 0.15 mg/mL EGCG solution was less stable, showing a gradual decrease in concentration and a maximal decrease of about 20–30% over 48 h. During the first day, the 0.15 mg/mL and 0.10 mg/mL EGCG solutions showed similar level of degradation, while the 0.05 mg/mL EGCG solution experienced fast degradation, and decreased by almost 80% over 48 h, which was similar to the final degradation of 0.10 mg/mL. In addition, during the first six hours, all the EGCG solutions except 0.05 mg/mL were stable, which suggests that EGCG aqueous solution stored without sunlight and air will not degrade over the short-term.

Oxygen in the air and sunlight seems appeared have great impacts on the stability of EGCG, especially in the low concentration groups. The storage of 0.05 mg/mL and 0.1 mg/mL EGCG solution under condition (D) led to 100% degradation. A similar result was observed for the 0.15 mg/mL EGCG solution, which degraded by 30–40% under condition (A), and almost 80% under condition (D). However, the 2 mg/mL EGCG solution was much more stable, even under condition (D), which suggests the presence of concentration-dependent stability at room temperature.

Based on these results, the ideal safe storage conditions for EGCG aqueous solutions include a concentration above 0.15 mg/mL without air and sunlight for one day. If the duration of storage exceeds one day, the longer the storage time, the higher the concentration required. In addition, it was shown that the 2.00 mg/mL EGCG solution could be stored for two days, and is thus a suitable concentration for storage.

The residual concentration of EGCG in EGCG aqueous solution stored with or without sunlight and air for different time periods is showed in Table 3. For all four concentrations, the stability dropped from condition (A) to condition (D), suggesting that the presence of both sunlight and oxygen can decrease the stability of EGCG. As for the 0.05 mg/mL EGCG solution, after being exposed to oxygen for 36 h, EGCG almost completely degraded and it could not be detected. In addition, EGCG could not be detected when it was exposed to oxygen for 48 h in the 0.10 mg/mL EGCG solutions. However, EGCG was detected during 48 h of storage in the 0.15 and 2.00 mg/mL EGCG solutions When the degradation level was reported as a percentage, rather than mg/mL, as in Figure 2 and Figure 3, the 2.00 mg/mL EGCG solution appeared to be rather stable. However, the 2.00 mg/mL EGCG solution still decreased to 1.786 ± 0.060 mg/mL under condition (D); thus, although it only decreased by about 20%, this was equivalent to about 0.2 mg/mL. Therefore, it was not a surprise to find that the 0.05, 0.10 EGCG solutions have completely degraded.

With respect to the speed of degradation, the trends of conditions (A) and (B) were only slightly different, and conditions (C) and (D) were similar as well. In other words, both EGCG solutions under conditions (A) and (B) had similar level of degradation, under conditions (C) and (D) also had similar level of degradation. This may suggest that oxygen has a more pronounced effect on the stability of EGCG than sunlight. In order to confirm the correlation between the stability of EGCG and effect factors, a correlation analysis was performed, and the Pearson correlation coefficients are shown in Table 4. It was not surprising to find that sunlight, oxygen and time all had negative effects on the residual concentration of EGCG, while concentration had a positive effect, which was also demonstrated that the stability of EGCG increased with an increasing concentration. As for sunlight, although the *p*-value was 0.425, its negative effect on EGCG actually existed. The correlation coefficient of oxygen was −0.266 with a *p*-value was 0.017, which indicates statistical significance. On the other hand, the correlation index of oxygen was larger than that of sunlight, proving the oxygen has a stronger effect on decreasing stability of EGCG than sunlight.

### 2.3. Analysis of Stability of EGCG Solution Atomized by the Ultrasonic Dental Scaler

An ultrasonic dental scaler consists of an ultrasonic generator and a transducer. The transducer converts the electromagnetic oscillations generated by the generator into vibrations that generate high-frequency oscillations. The EGCG aqueous solution was atomized through the work tip in order to determine whether ultrasound impacts the stability of EGCG. It is possible that the atomized EGCG solution may have extreme contact with air and sunlight over a short period of time. Table 5 shows the results of different concentrations of EGCG solution atomized into the vacuous bottle. None of the four concentrations showed significant difference between the control group, no matter whether sunlight was present or not. Although a slight decrease was observed at the lowest concentration (0.05 mg/mL), the *p*-value was still over 0.05. These results demonstrated that ultrasonic vibration would not make EGCG degrade, and ultrasound may not decrease the stability of EGCG in the short-term without oxygen.

Table 6 shows the results of the residual concentration of EGCG in the EGCG solution atomized by ultrasonic scaler with oxygen. Oxygen and sunlight appeared to have greater effects on the atomized EGCG solution than the EGCG solution under storage conditions (Table 4). In regard to the high concentration solution, regardless of whether it was exposed to sunlight or not, its concentration di not significantly degrade. Although the 2.00 mg/mL EGCG solution exposed to sunlight degraded slightly, the *p*-value was 0.094, indicating no statistical significance. However, when the initial concentration reduced, the stability of EGCG significantly decreased. The 0.15 mg/mL and 0.10 mg/mL EGCG solution atomized under sunlight showed significantly degradation (0.138 ± 0.004 mg/mL, *p* = 0.04; 0.084 ± 0.005 mg/mL, *p* = 0.028, respectively). As the concentration of EGCG was down to 0.05 mg/mL, the EGCG degraded significantly, even without sunlight (0.036 ± 0.001 mg/mL, *p* = 0.001). When exposed to sunlight, it decreased more (0.033 ± 0.001 mg/mL, *p* < 0.001). On the other hand, these results confirmed that the stability of EGCG in water depends on its initial concentration.

In order to evaluate whether sunlight affects the stability of the atomized EGCG solution, an intra-group *t*-test was performed between the groups with and without sunlight at the same concentration. Only the lowest concentration (0.05 mg/mL) had a significant difference, which demonstrates that during the short period of EGCG solution atomization by the ultrasonic dental scaler, sunlight does have a slight impact on the stability of EGCG, while oxygen in the air plays an important role in the degradation of EGCG. The atomized EGCG aqueous solution was less stable than the EGCG solution under storage conditions, and this is probably because when the solution was atomized into a mist, EGCG would have had extreme contact with oxygen and sunlight, which may have accelerated the degradation speed of EGCG. In other words, ultrasound may promote the degradation of EGCG through oxygen and sunlight, and the ultrasonic vibration itself may not breakdown the structure of EGCG as EGCG in aqueous solution atomized without oxygen did not significantly degrade.

## 3. Discussion

This is the first study to investigate whether ultrasound had an effect on the stability of EGCG. In this study, an ultrasonic dental scaler was used to atomize the EGCG aqueous solution, so EGCG may have extreme contact with air and sunlight over a short time period. Without oxygen, EGCG in aqueous solution was rather stable, even when it was atomized into a mist, which demonstrates that ultrasonic vibration itself does not make EGCG degrade. However, when the EGCG aqueous solution was atomized into air, EGCG in solution appeared less stable than under the storage conditions. The EGCG aqueous solution in the control group in this part of the study was exposed to oxygen and sunlight in the short-term, and it was stable, while the degradation of atomized EGCG aqueous solution with oxygen was significantly higher. These results demonstrate that ultrasound may accelerate the degradation of EGCG because of the extreme contact with oxygen and sunlight, rather than because of the ultrasonic vibration. In addition, the high concentration of EGCG (2.00 mg/mL) was still stable even under ultrasonic conditions. Thus, the use of EGCG aqueous solution through an ultrasonic dental scaler to treat periodontal infection may be feasible, because EGCG would not obviously decrease even when subjected to the ultrasonic conditions. Meanwhile, EGCG aqueous solution could actually reach the infected area and exercise its antimicrobial capacity through the work tip of the ultrasonic dental scaler, providing a new method for dentists to control the periodontal infection and may potentially improving treatment outcomes.

On the other hand, previous studies have proven that sunlight and oxygen affected the stability of EGCG [12,13,15,16], but we are the first to compare the strength of these two factors’ effects on EGCG in aqueous solution. Both parts of the study showed that, whether exposed to sunlight or not, the degradation level of EGCG did not change a lot, while EGCG decreased much more following exposure to oxygen. The Pearson correlation coefficients also confirmed that oxygen has a more pronounced effect on the stability of EGCG than sunlight.

In addition, the present study also has demonstrated that the stability of EGCG is concentration-dependent in water at room temperature, and the higher the initial concentration of EGCG is, the more stable it will be. This result is accordance with the previous studies, which showed that the initial concentration of EGCG in aqueous solution is an important factor for stability—a clear difference was shown, between the µM and mM range [16,22,35]. However, the reason for this phenomenon is not clear et al. showed that the rates of oxidation and epimerization reactions increased at the low concentrations of EGCG aqueous solution. As more water was present in these solutions, the pH, molecular mobility, and dissolved oxygen levels were greater, while at high concentrations of EGCG aqueous solution, the pH, molecular mobility, and dissolved oxygen levels decreased, so the rates of the two reactions were inhibited [36]. Thus, we propose that the oxidation and epimerization contributed to this phenomenon. Further study is needed to better explain the differences in the reaction mechanisms between the high concentration and low concentration solutions of EGCG.

However, the previous studies suggested that 1% EGCG exposed to a solar simulator emission would degrade almost 70% in an hour [12,13], while the 2.00 mg/mL EGCG aqueous solution in the present study was rather stable. This may relate to the formulation of EGCG. In those studies, the formulation of EGCG was an emulsion, while aqueous solution was used in the present study. In addition, the illumination in those studies was much higher than that in this study. On the other hand, the aim of those studies was to investigate the photodegradation of EGCG, but the effect of oxygen should be considered. As for the mechanism by which sunlight affects EGCG, the previous study suggested that the mechanism of EGCG photodegradation is different from the decomposition pathways involved in the catechin chemical instability [12]. The photochemical process was described in a previous review: The primary photochemical reaction is directly due to the absorption of a photon [37]. In other words, if there is a certain overlap between the absorption spectrum of the molecule and the incident radiation, radiation may provide energy to the molecule, which may be the reason why sunlight could accelerate the degradation process of EGCG. Thus, sunlight and oxygen both affect the stability of EGCG, and EGCG should be kept out of air and sunlight. Although EGCG is less stable in aqueous solution than in solid form, EGCG must be in water to be used with the ultrasonic dental scaler, which was why we investigated the stability of the EGCG aqueous solution in the present study.

Asah et al. [38] and Hirasaw et al. [39] reported that 0.50 mg/mL and 1.00 mg/mL EGCG solutions are the minimum inhibitory concentrations, respectively. However, Sang et al. [16] showed that both 0.50 mg/mL and 1.00 mg/mL EGCG aqueous solution were unstable, but 3.20 mg/mL EGCG aqueous solution is stable, so we aimed to find the minimum stable concentration. We found that the 2.00 mg/mL EGCG aqueous solution remained stable for two days, and we also aimed to cover the range of concentrations used in most animal studies in accordance with the study of Sang et al. [16]. Thus, the other four low concentrations were performed in the present study.

Interestingly, EGCG still degrades even if under conditions without sunlight and air, which suggests that EGCG degradation occurs regardless of auto-oxidation. This phenomenon may be explained by epimerization of EGCG to GCG. In the study of Sang et al. [16], when the auto-oxidation of EGCG was prevented, epimerization of EGCG to GCG became appreciable. GCG was the major product formed from EGCG at high concentrations, whereas with low concentrations of EGCG, EGCG dimers were formed mainly. The rates of these two reactions are affected by the level of oxygen, the concentration of EGCG, and the level of antioxidants [16]. However, the degradation products were not identified in the present study, and the further study was needed.

Although the EGCG aqueous solution was strictly kept out of sunlight or air in the present study, there were still some limitations. Regarding the isolation from oxygen, though the cap was tightened immediately, there was still some oxygen dissolved in the water. In addition, while EGCG was being measured in HPLC, sunlight was kept out, but it was impossible to keep air out of the HPLC machine. However, the present study showed that short time of air exposure had almost no impact on the stability of EGCG, which rules out the possibility that the existence of air could have interfered with the results during the HPLC analysis. On the other hand, when the EGCG aqueous solution was exposed to sunlight, sunlight may have influenced the temperature change. However, the whole study was performed in the laboratory, where the room temperature indoors was maintained at 25–28 °C, so the influence of sunlight on temperature was slight.

## 4. Materials and Methods

### 4.1. Materials

(−)-Epigallocatechin gallate (EGCG, 98% purity) was purchased from Yuanye Biotechnology Co. Ltd. (Shanghai, China). Mmembrane syringe filters (0.22 μm) were purchased from Dingguo Changsheng Biotechnology Co. Ltd. (Beijing, China). An ultrasonic dental scaler was from Guilin zhuomuniao Medical Devices Co. Ltd. (Guilin, China). A TA8120 digital luxmeter was purchased from Tasi Electronic Co. Ltd. (Suzhou, China). A MZ-2CNT diaphragm vacuum pump was from BRAND Trading Co. Ltd. (Shanghai, China) was used.

### 4.2. High Performance Liquid Chromatography (HPLC) Analysis

Analytical conditions. Prior to the analysis, all samples were filtered by 0.22 μm membrane syringe filters. Chromatographic analysis was performed on a Waters 2695 Series (Waters Technologies Shanghai Limited, Shanghai, China) LC system containing a quaternary pump, an online degasser, an autosampler, and a thermostatic column compartment set at 30 °C. Chromatographic separation was conducted on a Waters XSelect HSS T3 C18 column (4.6 mm × 250 mm, 5 μm). A gradient method was employed using mobile phase A consisting of distilled H_2_O, acetonitrile, acetic acid, and EDTA (888/90/20/2, *v*/*v*/*v*/*v*) and phase B consisting of distilled H_2_O, acetonitrile, acetic acid, and EDTA (178/800/20/2, *v*/*v*/*v*/*v*). The initial ratio is 100% A (10 min). Subsequently, the solvent composition was changed to 68/32 (A/B 15 min) and 100% A (10 min). The flow rate was 1 mL/min, and the injection volume was 10 μL. The UV detection wavelength was 278 nm [40].

Selectivity and Specificity. For the chromatographic method, developing a separation involves demonstrating specificity, which is the ability of the method to accurately measure the analyte response in the presence of all interferences. EGCG aqueous solution samples were prepared according to the procedures described above and analyzed.

Linearity. The calibration curve of HPLC method was evaluated by analyzing a series of standard EGCG aqueous solution samples at concentration from 0.005 to 2.5 mg/mL. The determination coefficient (R^2^) was calculated by means of the least-square analysis. The lower limit of quantification (LLOQ) is the lowest concentration of EGCG in a sample that can be determined with acceptable precision and accuracy.

Precision and Accuracy. Intra-day/inter-day accuracies and precisions of the method were evaluated by analyzing samples at 2 concentration levels in 4 replicates during a single day and in duplicate over 2 consecutive days. The precision was represented by relative standard deviation (RSD). The accuracies were determined by calculating the percentage of measured concentrations to the nominal concentrations of samples.

### 4.3. Stability of EGCG Aqueous Solution under Different Storage Conditions

The EGCG aqueous solution was prepared with distilled H_2_O. In order to prevent sunlight and air entering the solution, we used glass bottles wrapped up with tinfoil. After adding the pre-weighed EGCG power into the bottle, it was diluted with distilled H_2_O to full of bottle, and then the cap was tightened immediately. A series concentrations (0.05, 0.10, 0.15, 2.00 mg/mL) of EGCG aqueous solutions were prepared at room temperature (25–28 °C), with each concentration was divided into four conditions as follows: (A) exposed to neither sunlight nor air (tightened the cap of bottle and wrapped the bottle with tinfoil), (B) exposed to sunlight but not air (tightened the cap but did not wrap the bottle), (C) not exposed to sunlight but air (opened the cap and wrapped the bottle with tinfoil), and (D) exposed to sunlight and air (opened the cap but did not wrap the bottle). The diameter of the bottle mouth was about 1 cm, and the illumination intensity was about 300–500 lux. Each concentration of EGCG solution was stored under conditions A to D, respectively, for 6 h, 12 h, 24 h, 36 h, and 48 h, and the residual concentration of EGCG was measured with HPLC. The whole experiment was performed in the laboratory at room temperature (25–28 °C).

### 4.4. Stability of EGCG Aqueous Solution Atomized by the Ultrasonic Dental Scaler

A series of concentrations (0.05, 0.10, 0.15, 2.00 mg/mL) of EGCG aqueous solutions were prepared described in Section 2.3. The vibration frequency of the ultrasonic work tip was 42 kHz. Though the ultrasonic dental scaler, the EGCG solution was atomized into a mist. For the test group, each concentration of EGCG solution was atomized in a large glass bottle under the four conditions which were similar to Section 2.3: (a) exposed to neither air nor sunlight (inserted the tip into the vacuous bottle created by vacuum pump, and wrapped the bottle with tinfoil), (b) exposed to sunlight but not air (inserted the tip into the vacuous bottle created by vacuum pump, but did not wrap the bottle with tinfoil), (c) not exposed to sunlight but air (wrapped the bottle with tinfoil, but the bottle was not vacuous), (d) exposed to air and sunlight (the bottle was not wrapped with tinfoil, and the bottle was not vacuous), and the atomized solution was collected. The experiments were performed in the order of low to high concentrations, and there was a minimum period of 5 min between concentrations in which the next concentration of EGCG solution was run though the handpiece to wash out any excess of the last concentration of EGCG solution in the system.

For the control group, the EGCG aqueous solution was exposed to air and sunlight for the same amount of time as the test group in culture dishes, so that the EGCG solution was able to form a thin layer and have full contact with sunlight and air. The diameters of culture dishes were 10 cm.

### 4.5. Statistical Analysis

A group *t*-test was used to compare the significance of the differences between the groups of residual EGCG in EGCG aqueous solutions atomized by ultrasound, and a *p*-value < 0.05 was accepted as significant. Data are expressed as the mean value ± standard deviation (mean ± SD) (*n* = 3).

## 5. Conclusions

It is well known that EGCG has various benefits to health and great potential for medical applications. The present study was a preliminarily examination of its stability under storage and ultrasonic conditions. The limitation of the present study is that temperature, pH and ionic strength were not involved. In general, EGCG was shown to be highly unstable in water at room temperature, and it was more susceptible to oxygen in air than sunlight. Thus, more effort should be paid to the isolation of EGCG from air during storage. Moreover, the stability of EGCG dropped as its concentrations decreased; thus, it was shown to be concentration-dependent in water at room temperature. Ultrasound may accelerate the degradation of EGCG because EGCG in aqueous solution can have extreme contact with oxygen and sunlight, rather than because of the ultrasonic vibration. Thus, the EGCG aqueous solution has the potential to be used through an ultrasonic dental scaler to treat periodontal infection in the future.

## Figures and Tables

**Table 1:** Standard curve, correlation coefficient, linear ranges and lower limit of quantification (LLOQ) of EGCG in water.

Analyte	Calibration Curve	R^2^	Linear Range (mg/mL)	LLOQ (mg/mL)
EGCG	*Y* = 12215845.6*X* + 9542.815	0.999	0.005–2.5	0.005

**Table 2:** Intra-day/inter-day accuracy and precision of EGCG in water.

Nominal Concentration (mg/mL)	Intra-Day			Inter-Day		
	Measured Concentration (mg/mL)	RSD (%)	Accuracy (%)	Measured Concentration (mg/mL)	RSD (%)	Accuracy (%)
0.203	0.201 ± 0.003	1.73	98.88 ± 1.71	0.189 ± 0.008	4.19	93.14 ± 3.90
2.109	2.099 ± 0.040	1.93	99.51 ± 1.92	2.071 ± 0.040	1.93	99.85 ± 1.90

**Table 3:** The residual concentration of EGCG in the EGCG aqueous solution stored with or without sunlight and air for different time periods.

Initial Concentration (mg/mL)	Condition Name	Sunlight	Oxygen	Time (h)				
				6 h (mg/mL)	12 h (mg/mL)	24 h (mg/mL)	36 h (mg/mL)	48h (mg/mL)
0.05	(A)	No	No	0.038 ± 0.003	0.031 ± 0.004	0.015 ± 0.001	0.015 ± 0.001	0.012 ± 0.001
	(B)	Yes	No	0.036 ± 0.006	0.027 ± 0.001	0.013 ± 0.000	0.009 ± 0.001	0.008 ± 0.001
	(C)	No	Yes	0.033 ± 0.009	0.020 ± 0.006	0.006 ± 0.001	N	N
	(D)	Yes	Yes	0.032 ± 0.004	0.019 ± 0.002	0.005 ± 0.001	N	N
0.10	(A)	No	No	0.100 ± 0.006	0.090 ± 0.007	0.086 ± 0.005	0.070 ± 0.004	0.038 ± 0.002
	(B)	Yes	No	0.093 ± 0.005	0.088 ± 0.003	0.071 ± 0.003	0.065 ± 0.003	0.028 ± 0.001
	(C)	No	Yes	0.082 ± 0.004	0.074 ± 0.004	0.055 ± 0.005	0.041 ± 0.002	N
	(D)	Yes	Yes	0.082 ± 0.009	0.068 ± 0.008	0.044 ± 0.007	0.030 ± 0.004	N
0.15	(A)	No	No	0.150 ± 0.011	0.141 ± 0.004	0.135 ± 0.003	0.104 ± 0.003	0.093 ± 0.006
	(B)	Yes	No	0.136 ± 0.005	0.136 ± 0.007	0.118 ± 0.004	0.089 ± 0.005	0.087 ± 0.001
	(C)	No	Yes	0.128 ± 0.007	0.121 ± 0.009	0.107 ± 0.002	0.072 ± 0.006	0.058 ± 0.003
	(D)	Yes	Yes	0.128 ± 0.010	0.109 ± 0.004	0.070 ± 0.003	0.068 ± 0.010	0.029 ± 0.002
2.00	(A)	No	No	2.015 ± 0.031	2.000 ± 0.020	2.000 ± 0.040	1.998 ± 0.025	1.997 ± 0.031
	(B)	Yes	No	2.004 ± 0.041	2.001 ± 0.039	2.000 ± 0.028	1.999 ± 0.050	1.997 ± 0.056
	(C)	No	Yes	2.003 ± 0.024	2.002 ± 0.018	2.000 ± 0.022	1.997 ± 0.046	1.991 ± 0.039
	(D)	Yes	Yes	1.947 ± 0.010	1.900 ± 0.028	1.893 ± 0.032	1.806 ± 0.036	1.786 ± 0.060

N: not detected.

**Table 4:** Pearson correlation coefficient between the percentage of the residual concentration of EGCG and effect factors.

Effect Factor	Correlation Coefficient	*p*-Value
Sunlight	–0.091	0.425
Oxygen	–0.266	0.017 *
Time	–0.522	0.000 *
Concentration	0.706	0.000 *

* *p*-value < 0.05. Correlation coefficient: If the correlation coefficient is below 0, it means that the factor has a negative effect on the stabilization of EGCG; if the correlation coefficient is over 0, it means that the factor has a positive effect on the stabilization of EGCG.

**Table 5:** EGCG degradation in EGCG solution atomized by ultrasonic scaler without oxygen: A between-group analysis.

Initial Concentration (mg/mL)	Sunlight	Oxygen	Test Group Concentration (mg/mL)	Control Group Concentration (mg/mL)	*p*-Value
0.05	No	No	0.047 ± 0.002	0.052 ± 0.002	0.057
	Yes	No	0.046 ± 0.005		0.224
0.10	No	No	0.099 ± 0.003	0.099 ± 0.002	0.803
	Yes	No	0.099 ± 0.009		0.990
0.15	No	No	0.151 ± 0.002	0.149 ± 0.002	0.230
	Yes	No	0.150 ± 0.001		0.157
2.00	No	No	2.010 ± 0.018	2.003 ± 0.040	0.592
	Yes	No	2.005 ± 0.014		0.864

**Table 6:** The residual concentration of EGCG in the EGCG solution atomized by an ultrasonic scaler with oxygen: intra-group and between group analysis.

Initial Concentration (mg/mL)	Sunlight	Oxygen	Test Group Concentration (mg/mL)	Intra-Group *p*-Value	Control Group Concentration (mg/mL)	Between Group *p*-Value
0.05	No	Yes	0.036 ± 0.001	0.010*	0.052 ± 0.002	0.001 *
	Yes	Yes	0.033 ± 0.001			<0.001 *
0.10	No	Yes	0.098 ± 0.009	0.484	0.099 ± 0.002	0.879
	Yes	Yes	0.084 ± 0.005			0.028 *
0.15	No	Yes	0.146 ± 0.002	0.053	0.099 ± 0.002	0.117
	Yes	Yes	0.138 ± 0.004			0.040 *
2.00	No	Yes	1.997 ± 0.049	0.882	2.003 ± 0.040	0.875
	Yes	Yes	1.944 ± 0.032			0.094

* *p*-value < 0.05.
